# Supplementary material for: Mitochondrial Hyperactivity and Reactive Oxygen Species Drive Innate Immunity to the Yellow Fever Virus-17D Live-Attenuated Vaccine
Source: bioRxiv. 2024 Sep 15:2024.09.04.611167. Originally published 2024 Sep 7. Preprint. [Version 2] doi: 10.1101/2024.09.04.611167 (PMC11398391; doi:10.1101/2024.09.04.611167)
Supplement: 1 — Supplemental Figure 1: Differential mitochondrial morphodynamics are consistent across multiple cell types A Growth curve quantifying the viral titer and ELISA quantification of IFNβ secreted by Huh7 cells infected with YFV-17D (MOI 0.1) or DENV2 (MOI 0.1) over a 72 hour period. B IF of mitochondrial morphology in Huh7 cells following infection with mock, DENV2 (MOI 1), YFV-17D (MOI 0.1), or YFV-Asibi (MOI 1) for 48 hours. Nuclei are stained with DAPI in blue, mitochondria are stained with TOM20 in green, and infected cells are stained with NS3 in red. Supplemental Figure 2: siRNA treatment results in knockdown of host proteins Western blot quantifying protein following siRNA treatment with A siMAVS, B siSTING, C siMFN2, and D siDRP1. Treatment with siRNA resulted in significant knockdown of the respective protein. Supplemental Figure 3: Very few metabolites are differentially abundant between YFV-17D and mock infected cells at 24 and 36 hpi. A and B Volcano plots showing the significantly differentially abundant metabolites between YFV-17D infected Hep G2 cells and mock-infected HepG2 cells at A 24hpi and B 36hpi. Supplemental Figure 4: YFV-17D upregulates glycolysis between 24 and 48 hpi. A Metabolomic heatmap comparing the abundance of metabolites from the glycolytic pathway, pentose-5-phosphate pathway, and nucleotides following infection with mock, YFV-17D (MOI 0,1), YFV-Asibi (MOI 1), or DENV2 (MOI 1) in HepG2 cells. B Schematic of the glycolysis pathway with metabolites significantly downregulated in YFV-17D infection at 48hpi shown in blue and metabolites significantly upregulated in YFV-17D infection at 48hpi shown in red. C Schematic of the glycolysis and pentose-5-phosphate pathway with metabolites significantly downregulated in YFV-17D infection at 48hpi shown in in blue and metabolites significantly upregulated in YFV-17D infection at 48hpi shown in red. D Quantification of the media acidification rate obtained during the Seahorse XF mito stress test in [file NIHPP2024.09.04.611167V2-supplement-1.pdf]

bioRxiv preprint doi: https://doi.org/10.1101/2024.09.04.611167; this version posted September 15, 2024. The copyright holder for this preprint (which was not certified by peer review) is the author/funder. This article is a US Government work. It is not subject to copyright under 17 USC 105 and is also made available for use under a CC0 license.

Supplemental Figure 1

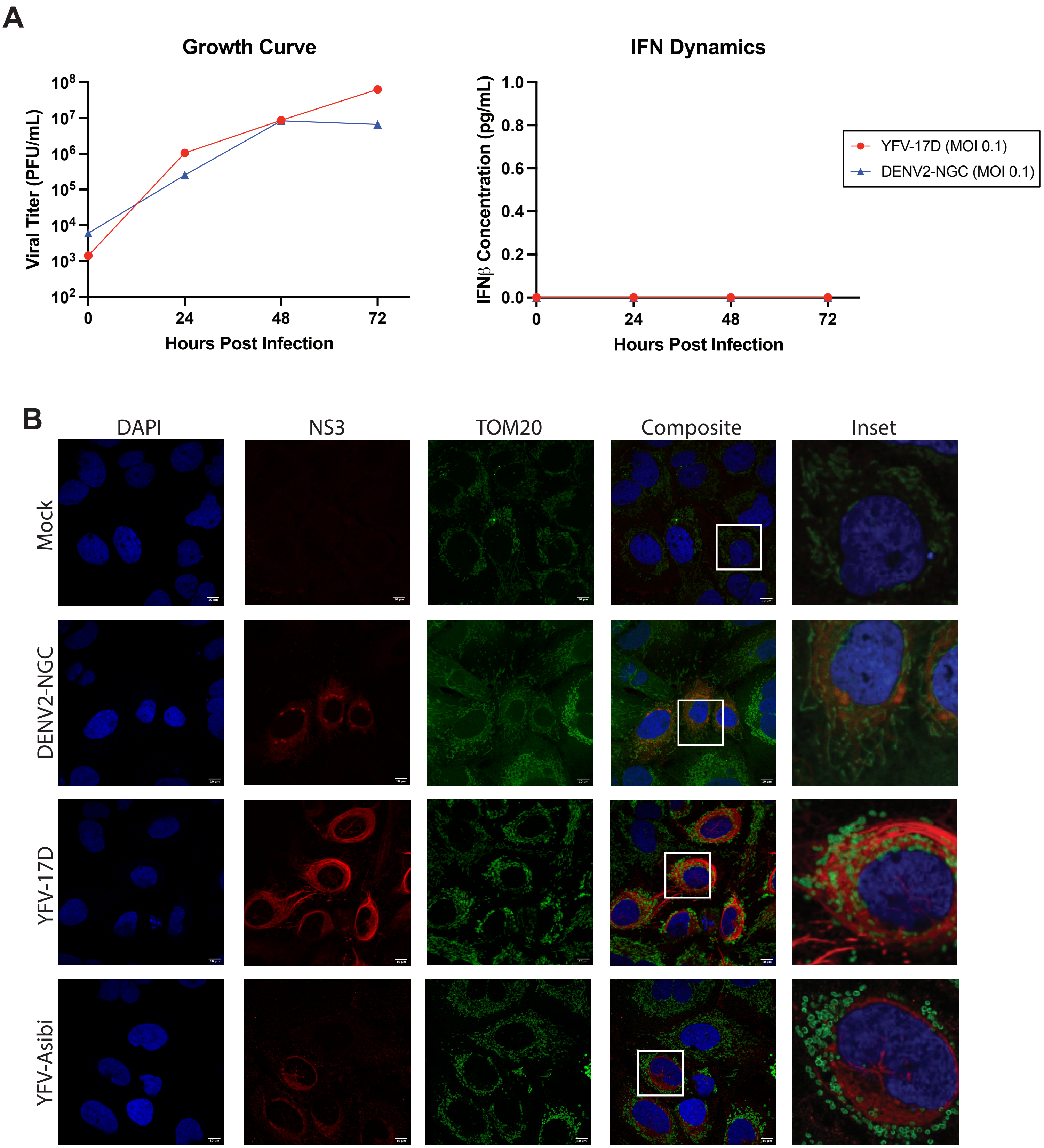

bioRxiv preprint doi: https://doi.org/10.1101/2024.09.04.611167; this version posted September 15, 2024. The copyright holder for this preprint (which was not certified by peer review) is the author/funder. This article is a US Government work. It is not subject to copyright under 17 USC 105 and is also made available for use under a CC0 license.

Supplemental Figure 2

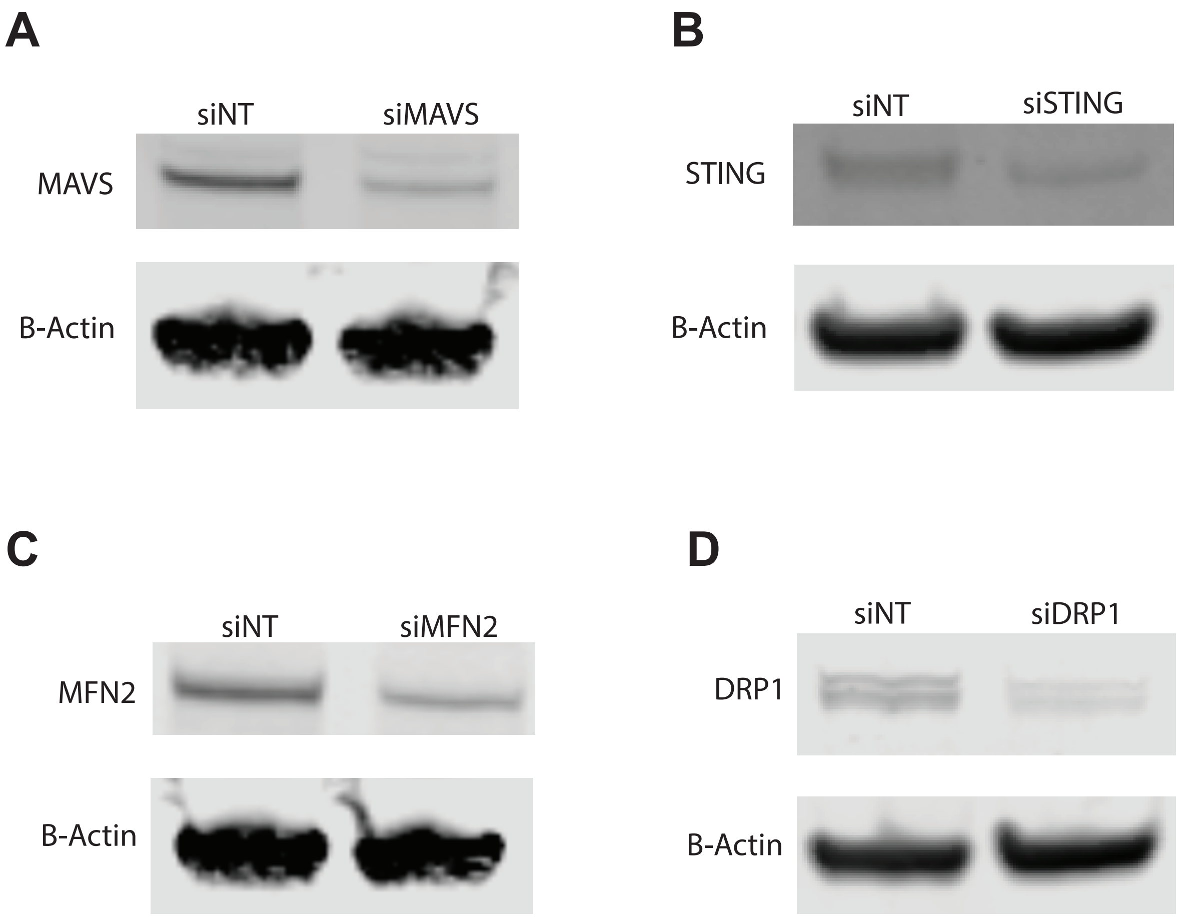

bioRxiv preprint doi: https://doi.org/10.1101/2024.09.04.611167; this version posted September 15, 2024. The copyright holder for this preprint (which was not certified by peer review) is the author/funder. This article is a US Government work. It is not subject to copyright under 17 USC 105 and is also made available for use under a CC0 license.

Supplemental Figure 3

A

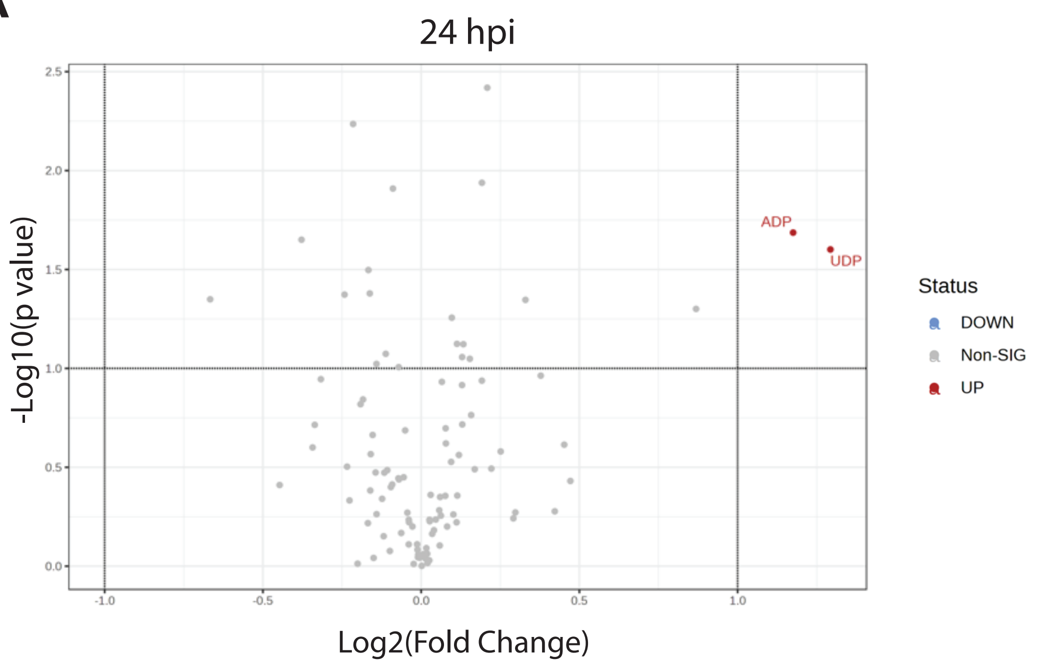

B

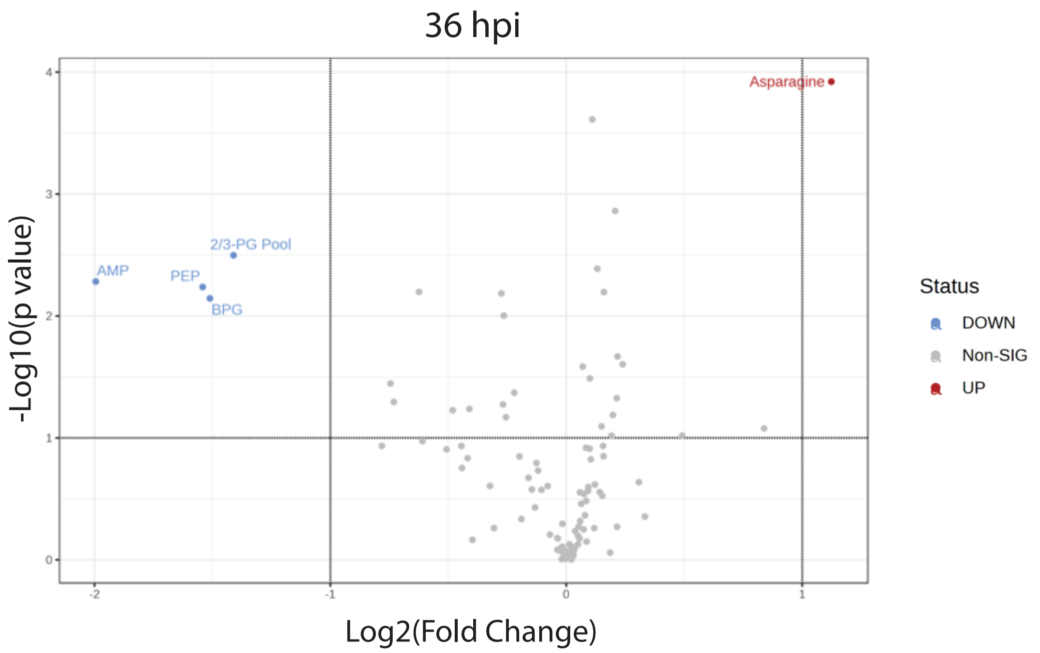

bioRxiv preprint doi: https://doi.org/10.1101/2024.09.04.611167; this version posted September 15, 2024. The copyright holder for this preprint (which was not certified by peer review) is the author/funder. This article is a US Government work. It is not subject to copyright under 17 USC 105 and is also made available for use under a CC0 license.

Supplemental Figure 4

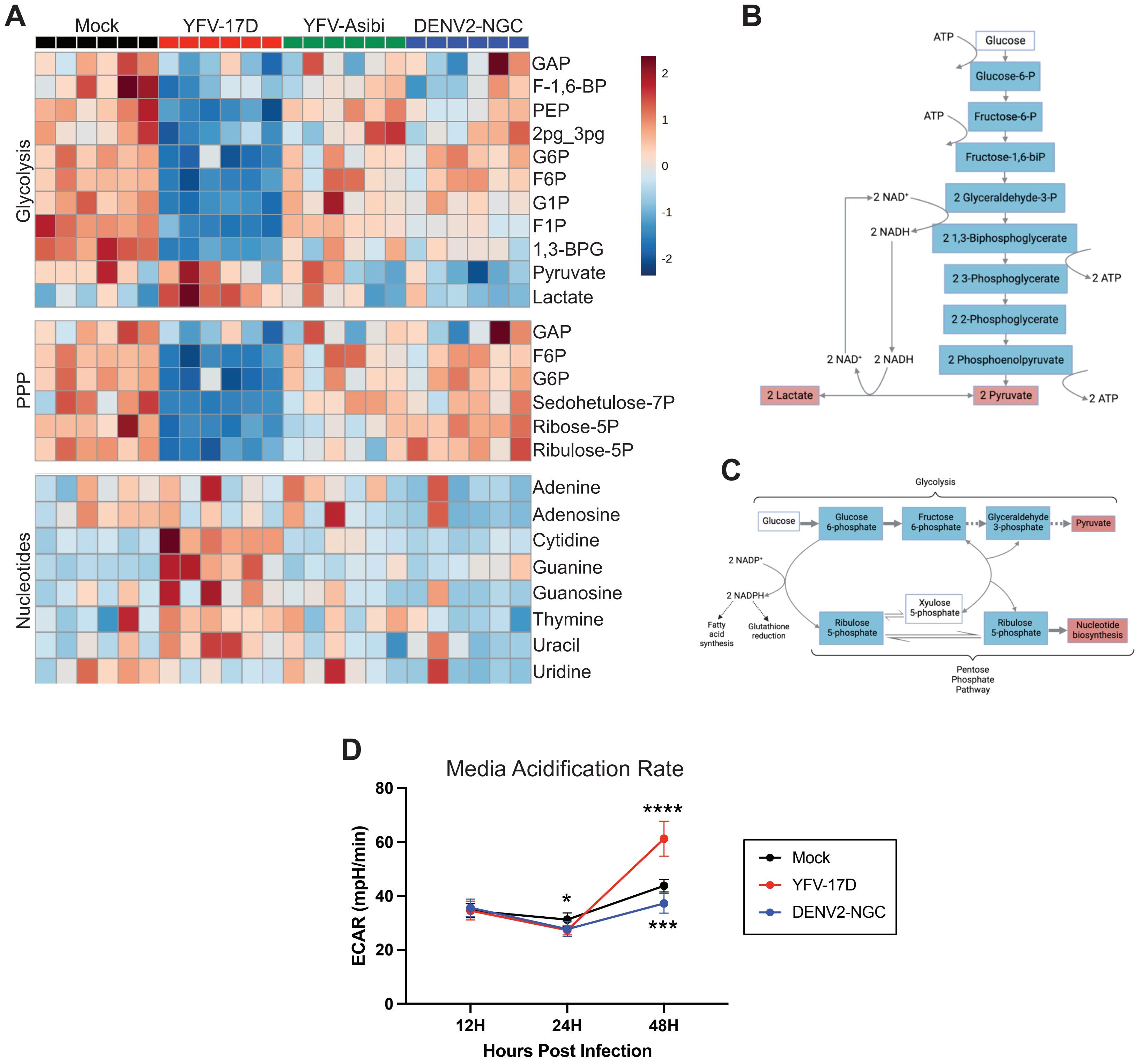

bioRxiv preprint doi: https://doi.org/10.1101/2024.09.04.611167; this version posted September 15, 2024. The copyright holder for this preprint (which was not certified by peer review) is the author/funder. This article is a US Government work. It is not subject to copyright under 17 USC 105 and is also made available for use under a CC0 license.

Supplemental Figure 5

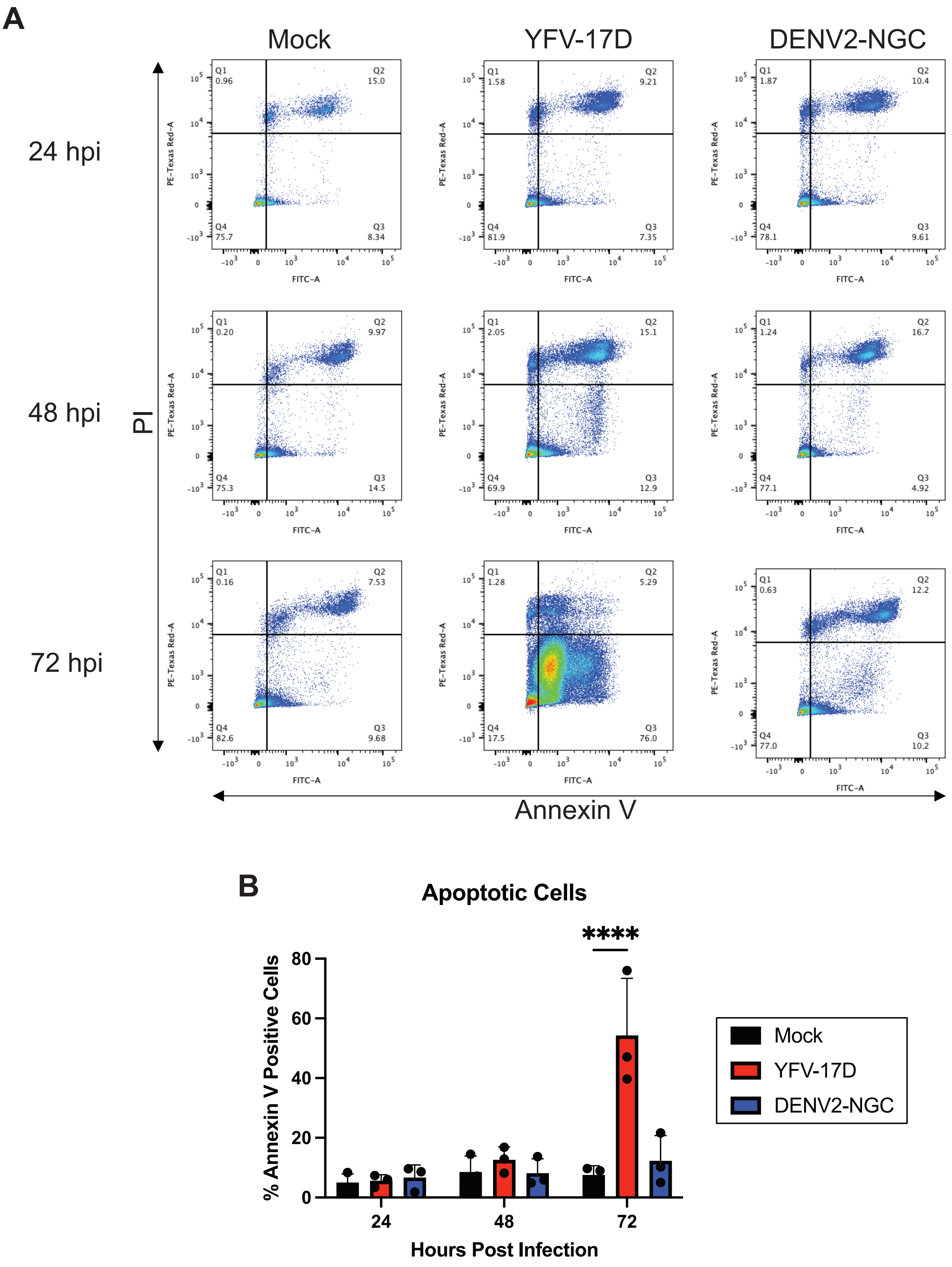

bioRxiv preprint doi: https://doi.org/10.1101/2024.09.04.611167; this version posted September 15, 2024. The copyright holder for this preprint (which was not certified by peer review) is the author/funder. This article is a US Government work. It is not subject to copyright under 17 USC 105 and is also made available for use under a CC0 license.

Supplemental Figure 6

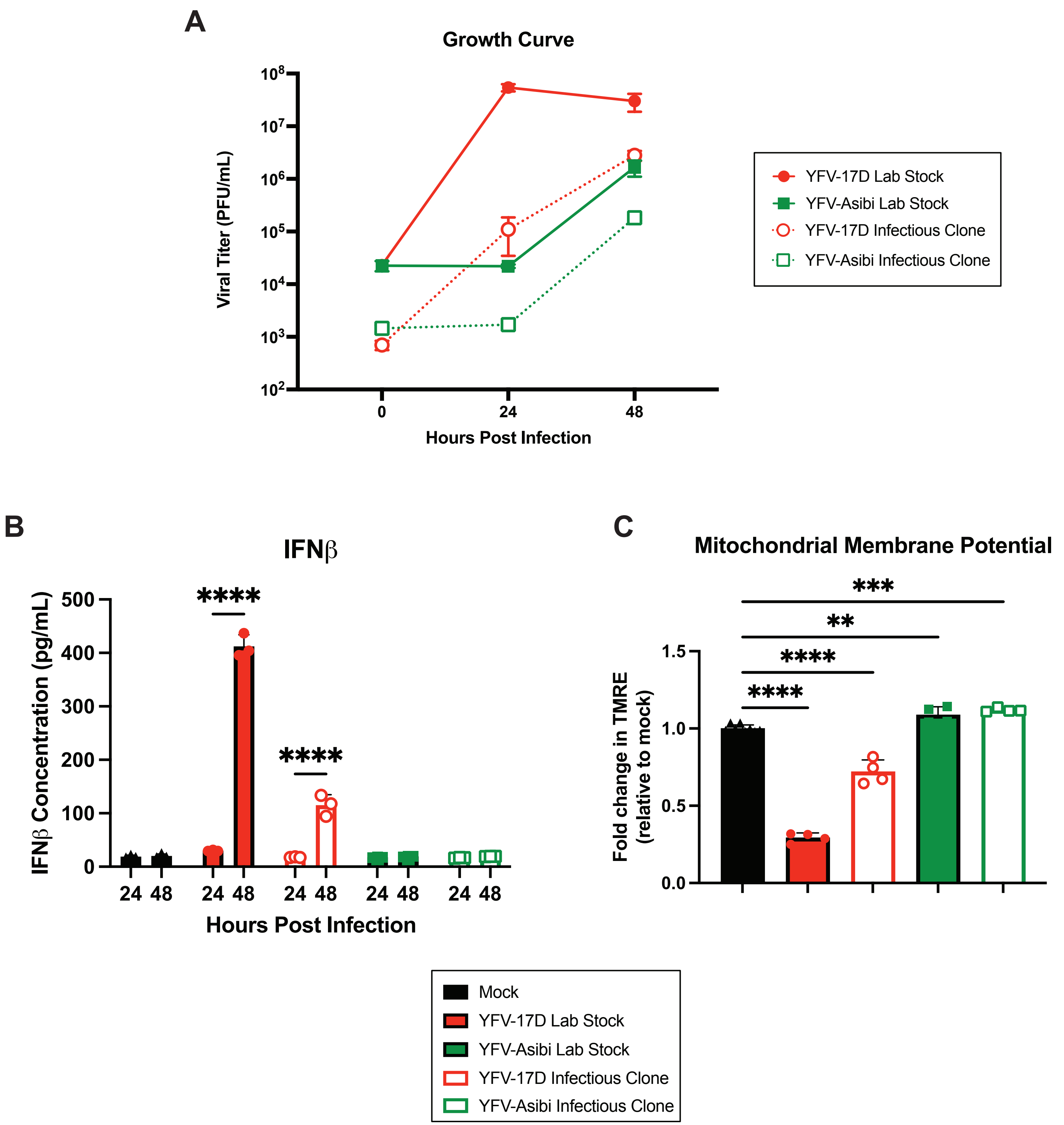

bioRxiv preprint doi: https://doi.org/10.1101/2024.09.04.611167; this version posted September 15, 2024. The copyright holder for this preprint (which was not certified by peer review) is the author/funder. This article is a US Government work. It is not subject to copyright under 17 USC 105 and is also made available for use under a CC0 license.

Supplemental Figure 7

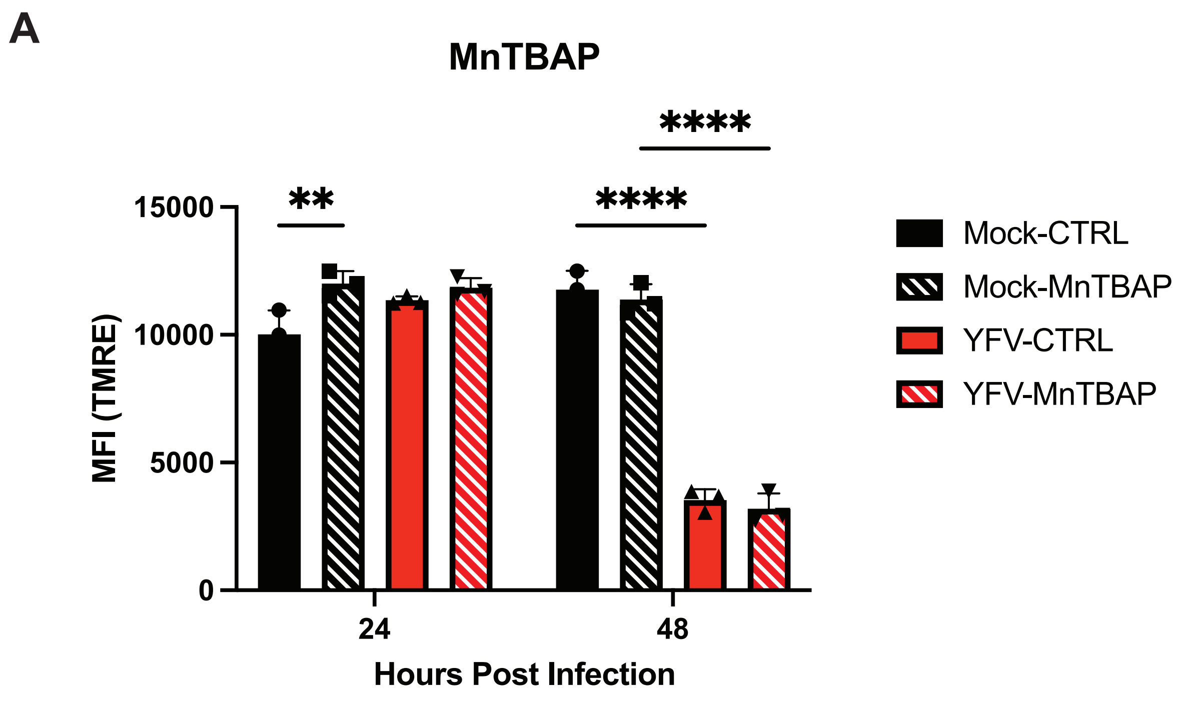

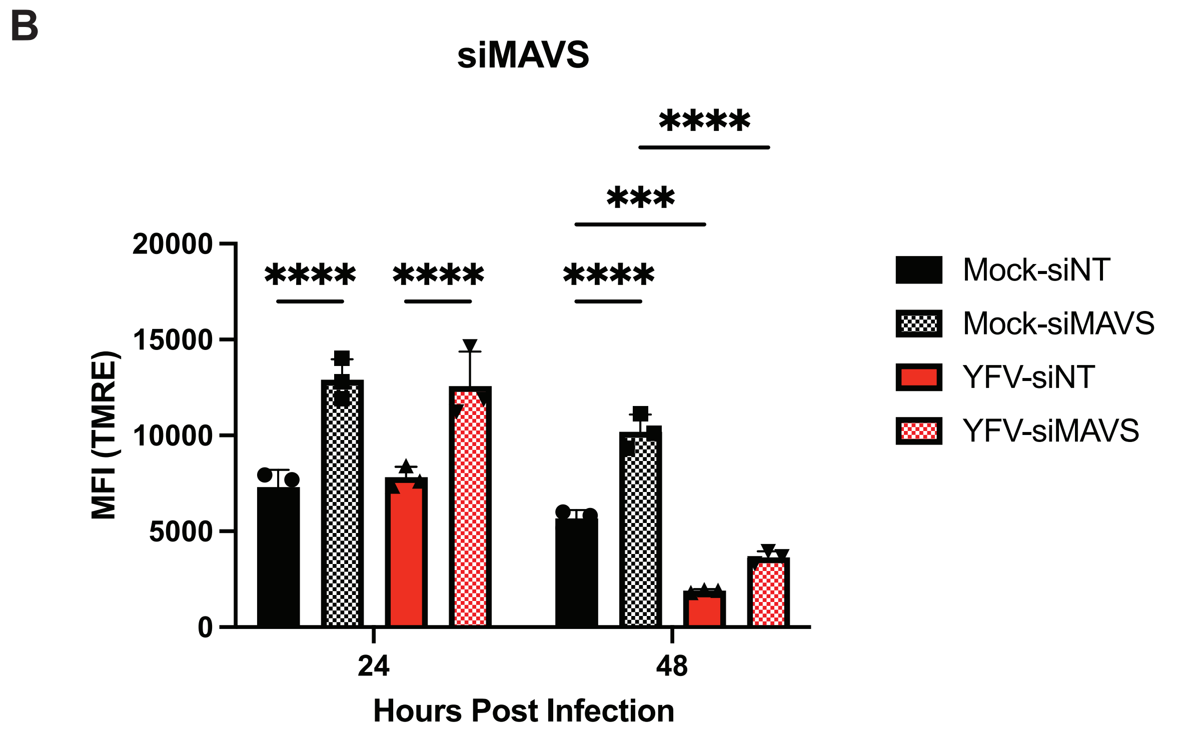

bioRxiv preprint doi: https://doi.org/10.1101/2024.09.04.611167; this version posted September 15, 2024. The copyright holder for this preprint (which was not certified by peer review) is the author/funder. This article is a US Government work. It is not subject to copyright under 17 USC 105 and is also made available for use under a CC0 license.

Supplemental Figure 8

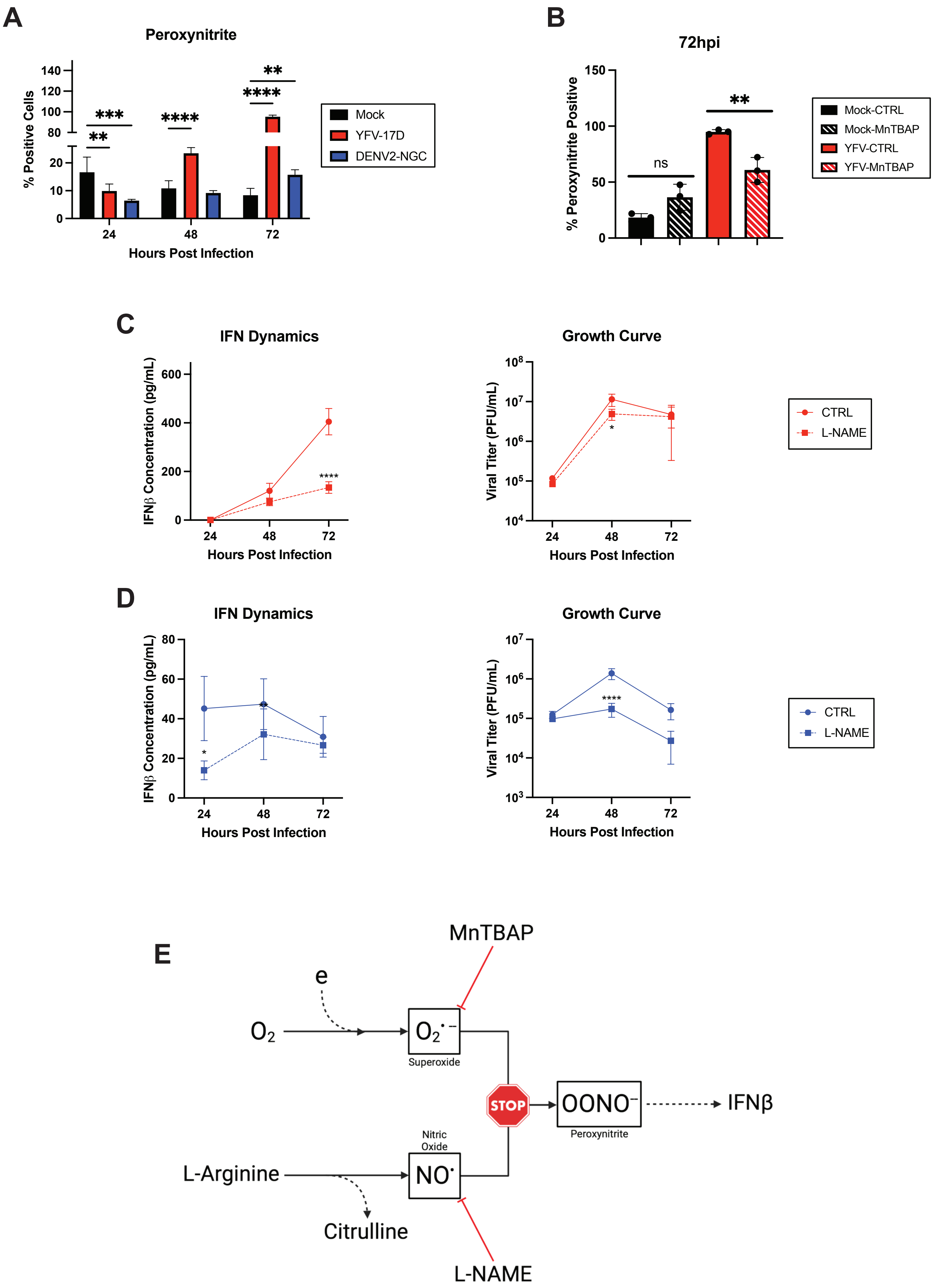

bioRxiv preprint doi: https://doi.org/10.1101/2024.09.04.611167; this version posted September 15, 2024. The copyright holder for this preprint (which was not certified by peer review) is the author/funder. This article is a US Government work. It is not subject to copyright under 17 USC 105 and is also made available for use under a CC0 license.

Supplemental Figure 9

A

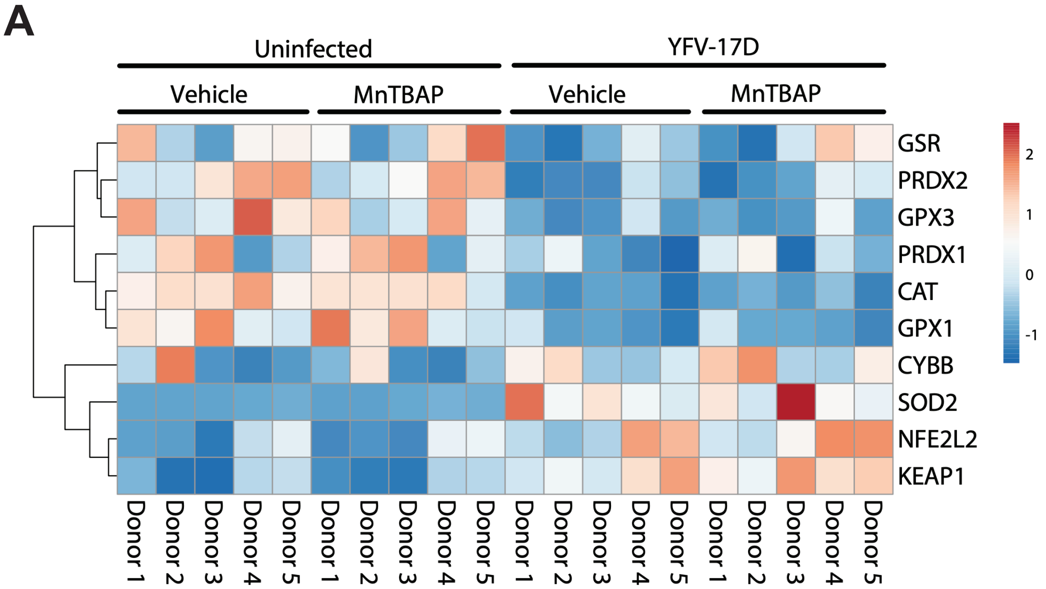

bioRxiv preprint doi: https://doi.org/10.1101/2024.09.04.611167; this version posted September 15, 2024. The copyright holder for this preprint (which was not certified by peer review) is the author/funder. This article is a US Government work. It is not subject to copyright under 17 USC 105 and is also made available for use under a CC0 license.

Supplemental Table S1

Pathway	Enrichment Score	P-Value	Adjusted P-Value	Leading Edge Genes
TRAF6 mediated IRF7 activation	-0.77	0.002	0.06	IFNA10;IFNA5;IFNA8;IFNA21;IFNA2; IRF7;IFNA7;IFNA14;SIKE1;IFNA1;TANK; TRAF2;TBK1;IFIH1;TRIM25;IFNB1;IKBKE; RNF135
Interferon alpha/beta signaling	-0.766	0.002	0.06	IFIT2;IFIT1;USP18;RSAD2;ISG20;IFNA10; ISG15;IFIT3;SOCS1;IFI27;OAS1;IFNA5; IRF2;IFNA8;IFITM1;IFITM2;IFNA21;IRF9; IFI35;IFNA2;OASL;IRF7;IFNA7;IFIT5; IFNA14;IFITM3;STAT2;STAT1;BST2;IRF8; IRF1;IFNA1;GBP2;MX1;IP6K2;MX2; HLA-B;PSMB8;OAS2
Regulation of IFNA/IFNB signaling	-0.722	0.002	0.06	USP18;IFNA10;SOCS1;IFNA5;IFNA8; IFNA21;IFNA2;IFNA7;IFNA14;STAT2; IFNA1
Interferon gamma signaling	-0.652	0.002	0.06	GBP5;MT2A;GBP4;GBP1;SOCS1;OAS1; IRF2;FCGR1A;TRIM6;TRIM31;IRF9; TRIM21;TRIM38;TRIM22;OASL;SP100; IRF7;TRIM5;IRF8;IRF1;GBP3;VCAM1; GBP2; TRIM35;IFI30;TRIM26; HLA-B;OAS2
Interferon signaling	-0.586	0.002	0.06	IFIT2;GBP5;IFIT1;USP18;MT2A;GBP4; RSAD2;ISG20;GBP1;IFNA10;ISG15;IFIT3; SOCS1;IFI27;KPNA5;OAS1;IFNA5;IRF2; IFNA8;FCGR1A;TRIM6;TRIM31;IFITM1; IFITM2;IFNA21;IRF9;TRIM21;DDX58; IFI35;IFNA2;TRIM38;UBE2L6;TRIM22; OASL;SP100;IRF7;IFNA7;IFIT5;IFNA14; IFITM3;TRIM5;STAT2;EIF4E3;STAT1;BST2; IRF8;IRF1;IFNA1;GBP3;VCAM1;GBP2; TRIM35;MX1;IFI30;TRIM26;IP6K2;MX2; NUP160;HLA-B;PSMB8;UBA7;OAS2
DDX58/IFIH1 mediated induction interferon alpha/beta	-0.574	0.002	0.06	IFNA10;ISG15;IFNA5;IFNA8;IFNA21; DDX58;IFNA2;UBE2L6;IRF7;IFNA7;FADD; IFNA14;DHX58;SIKE1;IFNA1;TANK; ATG12;TRAF2;UBA7;TBK1;IFIH1;TRIM25; HERC5;UBE2D3;IFNB1;IKBKE;RNF135; UBE2D1;RIPK1
