# Supplementary material for: Mitochondrial Hyperactivity and Reactive Oxygen Species Drive Innate Immunity to the Yellow Fever Virus-17D Live-Attenuated Vaccine
Source: bioRxiv. 2024 Sep 7:2024.09.04.611167. Preprint. [Version 1] doi: 10.1101/2024.09.04.611167 (PMC11398391; doi:10.1101/2024.09.04.611167)
Supplement: Supplementary file 1 [file NIHPP2024.09.04.611167v1-supplement-1.pdf]

bioRxiv preprint doi: https://doi.org/10.1101/2024.09.04.611167; this version posted September 7, 2024. The copyright holder for this preprint (which was not certified by peer review) is the author/funder. This article is a US Government work. It is not subject to copyright under 17 USC 105 and is also made available for use under a CC0 license.

Supplemental Figure 1: Differential mitochondrial morphodynamics are consistent across multiple cell types

A Growth curve quantifying the viral titer and ELISA quantification of IFNβ secreted by Huh7 cells infected with YFV-17D (MOI 0.1) or DENV2 (MOI 0.1) over a 72 hour period. **B** IF of mitochondrial morphology in Huh7 cells following infection with mock, DENV2 (MOI 1), YFV-17D (MOI 0.1), or YFV-Asibi (MOI 1) for 48 hours. Nuclei are stained with DAPI in blue, mitochondria are stained with TOM20 in green, and infected cells are stained with NS3 in red.

Supplemental Figure 2: siRNA treatment results in knockdown of host proteins

bioRxiv preprint doi: https://doi.org/10.1101/2024.09.04.611167; this version posted September 7, 2024. The copyright holder for this preprint (which was not certified by peer review) is the author/funder. This article is a US Government work. It is not subject to copyright under 17 USC 105 and is also made available for use under a CC0 license.

Western blot quantifying protein following siRNA treatment with **A** siMAVS, **B** siSTING, **C** siMFN2, and **D** siDRP1. Treatment with siRNA resulted in significant knockdown of the respective protein.

Supplemental Figure 3: Very few metabolites are differentially abundant between YFV-17D and mock infected cells at 24 and 36 hpi.

A and B Volcano plots showing the significantly differentially abundant metabolites between YFV-17D infected Hep G2 cells and mock-infected HepG2 cells at **A** 24hpi and **B** 36hpi.

Supplemental Figure 4: YFV-17D upregulates glycolysis between 24 and 48 hpi.

A Metabolomic heatmap comparing the abundance of metabolites from the glycolytic pathway, pentose-5-phosphate pathway, and nucleotides following infection with mock, YFV-17D (MOI 0,1), YFV-Asibi (MOI 1), or DENV2 (MOI 1) in HepG2 cells. **B** Schematic of the glycolysis pathway with metabolites significantly downregulated in YFV-17D infection at 48hpi shown in blue and metabolites significantly upregulated in YFV-17D infection at 48hpi shown in red. **C** Schematic of the glycolysis and pentose-5-phosphate pathway with metabolites significantly downregulated in YFV-17D infection at 48hpi shown in in blue and metabolites significantly upregulated in YFV-17D infection at 48hpi shown in red. **D** Quantification of the media acidification rate obtained during the Seahorse XF mito stress test in mock, YFV-17D, or DENV2 infected HepG2 cells over a 48 hour period. Values represent the mean ± SD (n=12). Statistical significance was assessed using two-way ANOVA followed by Dunnett's multiple comparisons test (D). * $p<0.05$, ** $p<0.01$, *** $p<0.001$, **** $p<0.0001$.

bioRxiv preprint doi: https://doi.org/10.1101/2024.09.04.611167; this version posted September 7, 2024. The copyright holder for this preprint (which was not certified by peer review) is the author/funder. This article is a US Government work. It is not subject to copyright under 17 USC 105 and is also made available for use under a CC0 license.

Supplemental Figure 5: YFV-17D does not induce apoptosis until late timepoints.

A Representative flow cytometry dot plots quantifying Annexin V and propidium iodide in mock, YFV-17D (MOI 0.1), and DENV2 (MOI 1) infected HepG2 cells at 24hpi, 48hpi, and 72hpi. **B** Quantification of the percentage of apoptotic cells in mock, YFV-17D, and DENV2 infected cells at 24hpi, 48hpi, and 72hpi. Values represent the mean ± SD (n=3). Statistical significance was assessed using two-way ANOVA followed by Dunnett's multiple comparisons test (D). **** $p<0.0001$.

Supplemental Figure 6: Infectious clones mirror the phenotypes of lab stock viruses

A Growth curve quantifying viral titer of HepG2 cells infected with YFV-17D (MOI 0.1) or YFV-Asibi (MOI 0.1) generated from lab stocks or infectious clones over a 48 hour period. **B** ELISA quantification of IFNβ secreted by HepG2 cells infected with lab stocks or infectious clones at 24 and 48 hpi. **C** Quantification of mitochondrial membrane potential (TMRE) in HepG2 cells infected with lab stock or infectious clone viruses at 48hpi. Values represent the mean ± SD (A, B: n=3; C: n=4). Statistical significance was assessed using two-way ANOVA followed by Dunnett's post hoc test for multiple groups (B) or ordinary one-way ANOVA followed by Sidak's post hoc test for multiple groups (C). ** $p<0.01$, *** $p<0.001$, **** $p<0.0001$.

Supplemental Figure 7: Loss of mitochondrial membrane potential is independent of type-I IFN signaling

bioRxiv preprint doi: https://doi.org/10.1101/2024.09.04.611167; this version posted September 7, 2024. The copyright holder for this preprint (which was not certified by peer review) is the author/funder. This article is a US Government work. It is not subject to copyright under 17 USC 105 and is also made available for use under a CC0 license.

Quantification of mitochondrial membrane potential (TMRE) in HepG2 cells infected with mock or YFV-17D (MOI 0.1) treated with **A** MnTBAP or **B** siMAVS to inhibit type-I IFN signaling. Values represent the mean ± SD (n=3). Statistical significance was assessed using two-way ANOVA followed by Tukey's post hoc test for multiple groups. ** p<0.01, *** p<0.001, **** p<0.0001.

Supplemental Figure 8: Peroxynitrite appears to be the main source of ROS in YFV-17D infection. **A** Quantification of peroxynitrite staining in mock, YFV-17D (MOI 0.1), and DENV2 (MOI 1) infected HepG2 cells at 24hpi, 48hpi, and 72hpi using flow cytometry. **B** Flow cytometric quantification of peroxynitrite staining in mock, YFV-17D, and DENV2 infected HepG2 cells treated with MnTBAP or a vehicle control for 48 hours. **C and D** Growth curve quantifying the viral titer and ELISA quantification of IFNβ secreted by HepG2 cells infected with **C.** YFV-17D or **D** DENV2 and treated with L-NAME [5mM] at 0hpi. **E** Schematic showing the proposed mechanism by which both MnTBAP and L-NAME inhibit peroxynititre formation therby blocking type-I IFN production. Values represent the mean ± SD (n=3). Statistical significance was assessed using two-way ANOVA followed by Dunnett's multiple comparisons test (A), ordinary one-way ANOVA followed by Tukey's multiple comparisons test (B), or two-way ANOVA followed by Sidak's multiple comparisons test (C, D). **** p<0.0001.

Supplemental Figure 9 :MnTBAP treatment ameliorates effects of YFV-17D infection. **A** Heatmap comparing the expression of oxidative stress genes in mock and YFV-17D (MOI 0.1) infected human DCs treated with and without MnTBAP.
